# A comparison of the larvivorous habits of exotic *Poecilia reticulata* and native *Aplocheilus parvus*

**DOI:** 10.1186/s12898-018-0180-1

**Published:** 2018-08-14

**Authors:** G. K. Achini W. Fernando, Sevvandi Jayakody, W. M. Hiranya K. Wijenayake, Gawrie N. L. Galappaththy, Mangala Yatawara, Jeevanie Harishchandra

**Affiliations:** 1 Department of Aquaculture and Fisheries, Wayamba University of Sri Lanka, Makandura, Gonawila Sri Lanka; 2 World Health Organization, Global Malaria Programme, Geneva, Switzerland; 3 Department of Zoology and Environment Management, University of Kelaniya, Kelaniya, Sri Lanka; 4 Anti Malaria Campaign, Colombo 05, Sri Lanka

**Keywords:** Aquatic ecosystem, Biological control, Diet composition, Fish

## Abstract

**Background:**

The exotic fish *Poecilia reticulata* is promoted in the tropics as a biological control agent for aquatic pathogenic carriers, such as mosquitoes. Such control measures are often adopted blindly, ignoring the potential of native species and the adverse effects of introduced species. The present study was conducted to assess the diet composition of two species of fish, the native *Aplocheilus parvus* and exotic *P. reticulata*, and to assess the availability of food items in their natural environment in four types of aquatic systems. Diet composition was estimated using 24 h gut contents analysis, in a clay quarry pit and a perennial reservoir for *A. parvus*, and in a man-made canal and a second-order natural stream for *P. reticulata.* Food items in these environments were quantified by analyzing water samples collected every 2 h.

**Results:**

The diet of *A. parvus* in the clay quarry pit and reservoir consisted of adult or larval stages of Insecta, Maxillopoda and Malacostraca. In both habitats, *A. parvus* selectively fed on insect parts and insect larvae. The diet of *P. reticulata* consisted of filamentous algae, diatoms and detritus. The diet of *A. parvus* showed active selection of insectivore food items against their low availability. In contrast, the diet of *P. reticulata* showed consumption of food items in accordance with their availability in the environment. The highest mean number of food items in the gut for *A. parvus* was recorded around mid-day in the clay quarry pit, but no peak feeding time was identified in the perennial reservoir. For *P. reticulata*, peak feeding was recorded around mid-day in both the habitats.

**Conclusion:**

Irrespective of the type of environment and rate of occurrence, *A. parvus* preferred insect and insect larvae, whereas *P. reticulata* consumed the most readily available food items. The active selection of insects by *A. parvus* suggests they may have value as a biological control agent.

**Electronic supplementary material:**

The online version of this article (10.1186/s12898-018-0180-1) contains supplementary material, which is available to authorized users.

## Background

Fish are being introduced to new areas as biological control agents [1] for insect-related vector-borne diseases [2, 3]. However, this practice is sometimes questioned due to damage to non-target organisms and ecosystem functioning [4, 5] and the invasiveness of exotics [6]. The species *Poecilia reticulata* is a known invasive [7, 8], but has been introduced to many parts of the world as a biological control agent [9, 10].

In many instances, the potential of native fish species for biological control has not being explored, although the practice is encouraged [11] and promising results have been obtained from a handful of studies [12–14]. Since the family Aplocheilidae are already recognized as predators and have been proven to control the larval stages of aquatic insects [15, 16], the diet composition of the native *Aplocheilus parvus* was compared with the exotic *P. reticulata* in some selected water bodies in Sri Lanka. *P. reticulata* has spread along rivers and other waterways to new locations [17] and can now be found in both lotic and lentic environments. *Aplocheilus parvus* is a surface dwelling fish species inhabiting lotic and lentic systems in wet, intermediate and dry zones of Sri Lanka, both in moderately saline and fresh waters, where *P. reticulata* has been introduced as biological control. However, the diet of *P. reticulata*, in relation to available food sources, has not been evaluated in these new habitats. The objective of the current study was, therefore, to evaluate the food choices of *P. reticulata* and *A. parvus* in the aquatic systems where they live.

## Methods

Diet composition for both species was determined by 24-h gut contents analysis [18]. Initially their presence in the different aquatic systems was assessed, in order to select appropriate sampling locations. Accordingly, diurnal and nocturnal surveys were carried out in an abandoned clay quarry pit (7°.3280′ N; 80°.0241′, E) and in a perennial reservoir (7°.1733 N; 79°.9619 E) for *A. parvus.* A man-made canal (7.1314°N; 79.8764°E) and a second-order natural stream (7.2268°N; 80.1958°E) were surveyed for *P. reticulata,* as they represent the main aquatic systems to which this species has been introduced. Sampling was carried out between 2012 and 2013 (*A. parvus*: January 2012 and May 2013, *P. reticulata*: April 2012 and June 2013). Prevailing weather prevented starting of sampling at the same time.

At each of 4 different sampling sites, samples of 12 fish consisting of both males and females of *A. parvus* (weight: $\bar{x}$ = 0.14 ± 0.004 g SD; total length: $\bar{x}$ = 25.5 ± 2.89 mm SD) and *P. reticulata* (weight: $\bar{x}$ = 0.2078 ± 0.1258 g SD; total length: $\bar{x}$ = 25.050 ± 4.334 mm SD) were collected at 2-h intervals for 24 h, resulting in a total of 144 fish per site. The fish were immediately transferred to a freshly prepared solution of 10% buffered formalin. A plankton net (110 μm mesh size and 26.4 cm in diameter) was dragged for 5 m at the water surface to collect natural food items and the contents were preserved in 5% buffered formalin and Lugol’s Iodine solution, for zooplankton and phytoplankton respectively, at every sampling [19, 20]. Later, all the specimens were blotted and weighed. Standard length and total length were measured and the fish were carefully dissected and their guts removed. For each fish, the length and weight of the gut were obtained. Gut fill (the fraction filled out of 10 parts) was recorded for each fish and the types of material in the gut were recorded using a Sedgewick Rafter Cell. A common copepod was taken as the arbitrary unit for *A. parvus.* Several copepods were selected and their length and width were recorded to calculate the area. After determining the mean area of a copepod, every food item identified were measured for length and width. The total area of a given food item was divided by the mean area of a copepod to determine the number [21, 22]. In *P. reticulata,* only the anterior part of the gut (the stomach and a small part of the foregut at the point where the gut turns 180°) was used in the analyses and a common diatom was used as the arbitrary unit. A total of 576 gut samples were analyzed with 144 samples per species per site.

Food particles in the water samples were analyzed separately for all four sites at each time period, with dilution where necessary, using 100 cells of a Sedgewick Rafter Cell. A common diatom was taken as the arbitrary unit.

Tukey’s mean separation was used to identify differences in gut fill. Interactions between sex, time The mean numbers of different types of food particles present in the gut, for each species, at each sampling time and at all four sites were calculated and then used to identify the times of peak gut fill for each sex of each species at each site. Analysis of variance (ANOVA) with, site and species, on the mean number of each food item present, were estimated by one-way and two-way ANOVA. Where the data were unbalanced, General Linear Models were used. The number of different types of food particles present in the gut and in the environment were converted into percentages of occurrence and the data were used to help understand the relationships between consumption and availability. Further, Electivity Index of Ivlev (1961) was calculated to find out the selectivity of food items against the availability in the environment.

### Ethical committee

Approval for collection and sacrifice of fish and other field work was obtained from the Faculty of Graduate Studies of University of Kelaniya, Sri Lanka and the Ethics Committee of the Wayamba University of Sri Lanka, in the Faculty of Livestock, Fisheries and Nutrition. Collection of *A. parvus* from the wild was done with the permission from Department of Wildlife Conservation, Sri Lanka.

## Results

### Diet of *A. parvus*

Food items detected in the clay quarry pit and reservoir environments consisted of filamentous algae, detritus, copepods, insect parts and other items, which included plant parts, eggs and other unidentified materials. While copepods and filamentous algae were the main food items in the clay quarry pit, detritus represented the highest percentage of food items in the reservoir.

The diet of *A. parvus* in the clay quarry pit and the reservoir consisted of adult or larval stages of the classes Insecta, Maxillopoda and Malacostraca. Both adult and larval stages of Coleopterans were detected in the gut of *A. parvus* in both environments, whilst Hymenopterans were detected only in the gut of *A. parvus* inhabiting the clay quarry pit. Additionally, food items such as plant parts, eggs and other unidentified materials were present in the gut at both the sites.

**Fig. 1:**
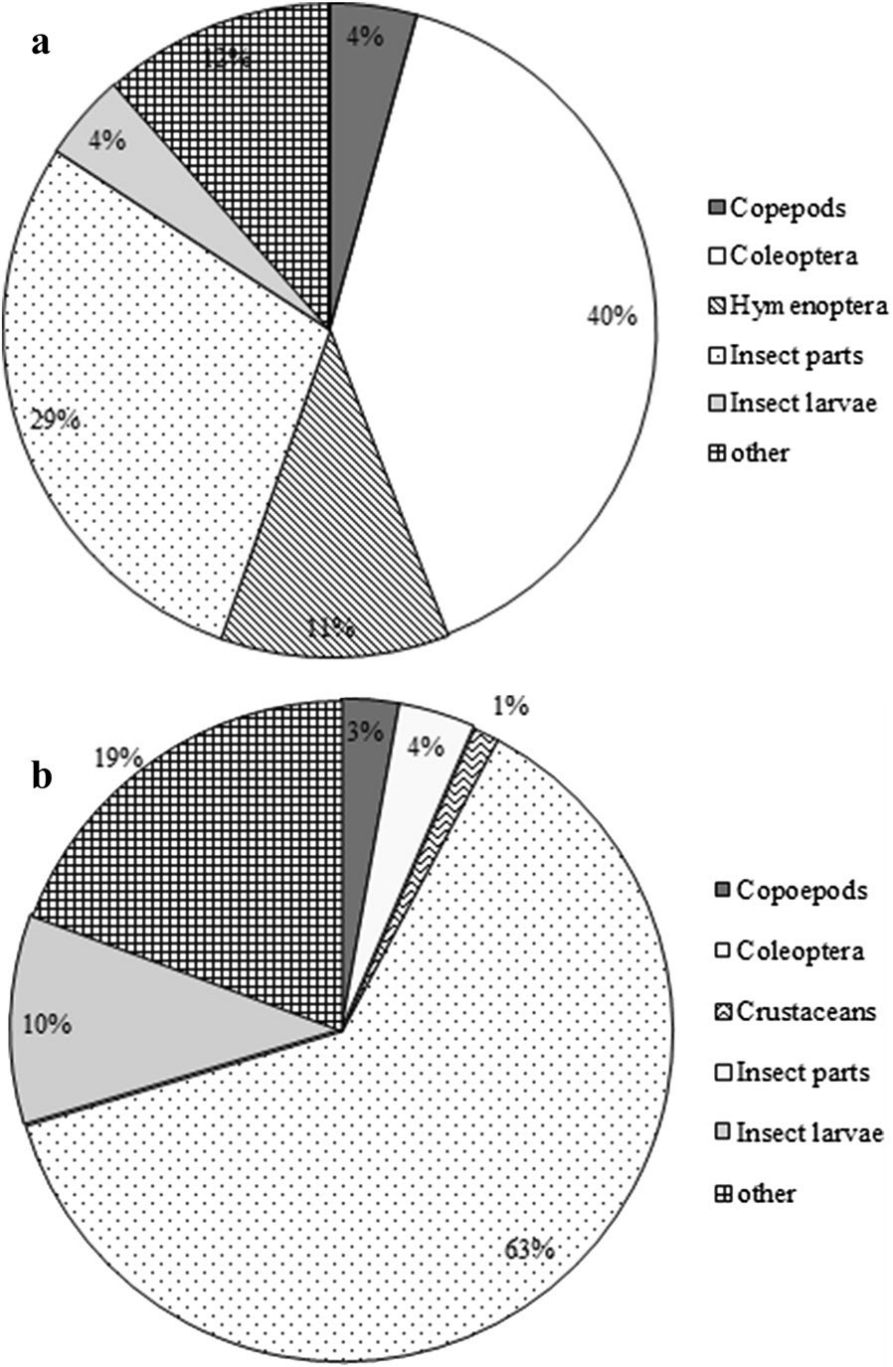
Percentage abundances of food items in the gut contents of *A. parvus* sampled in **a** clay quarry pit; **b** reservoir

Whilst Coleoptera were the main food item (40% of gut fill) of *A. parvus* in the clay quarry pit, unidentified insect parts were the main food item (63%) in the reservoir (Fig. 1a, b).

**Fig. 2:**
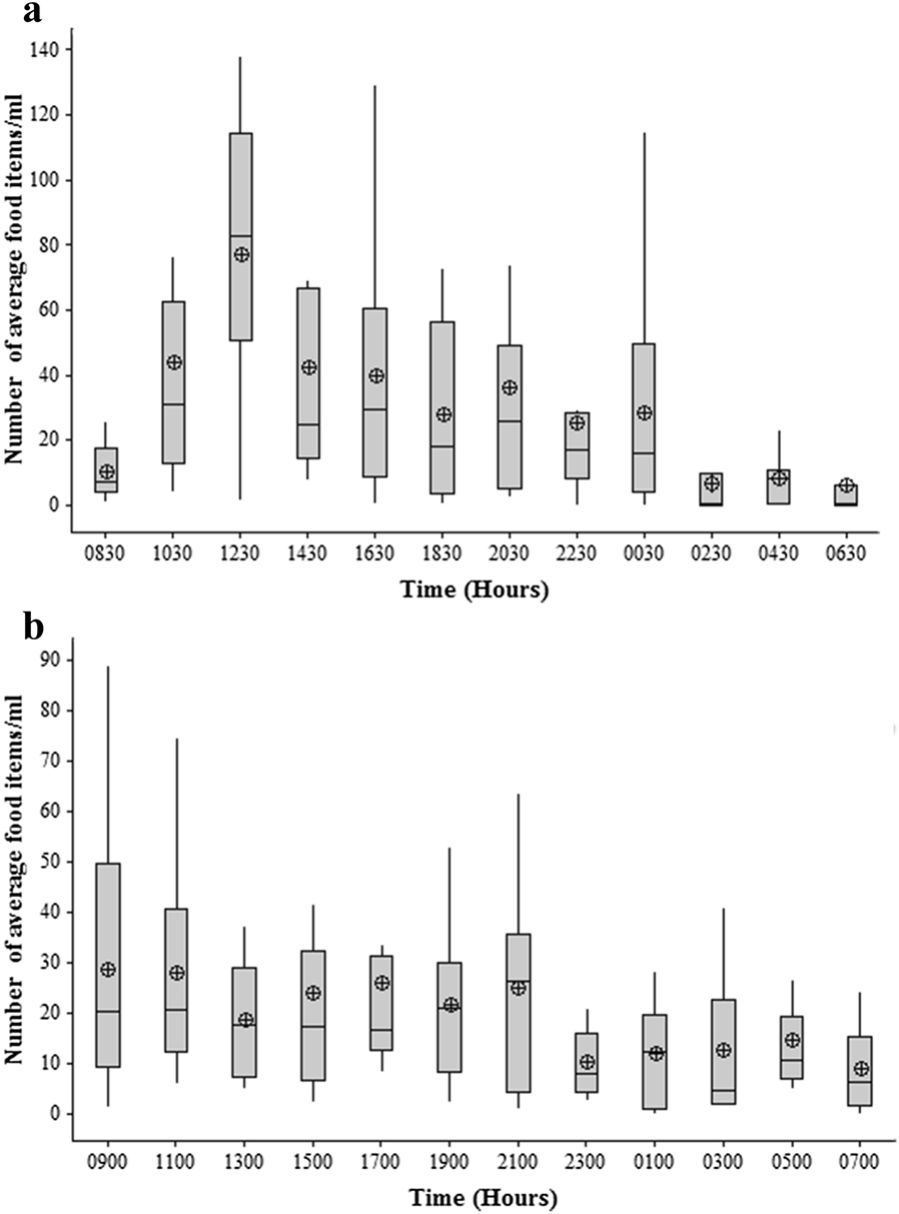
Fluctuations in number of mean food items with time in the gut contents of *A. parvus* sampled in **a** clay quarry pit and **b** reservoir. The box plot whiskers depict the range and circled crosses depict the mean number of food items at each time

The highest mean number of food items in the gut was detected at mid-day, around 1230 h, in the clay quarry pit (Fig. 2a) and the mean number of food items during that time was significantly higher than all other times (ANOVA, *df*_11,120_, P < 0.005). By contrast, in the reservoir, *A. parvus* did not show a peak feeding time (Fig. 2b).

**Fig. 3:**
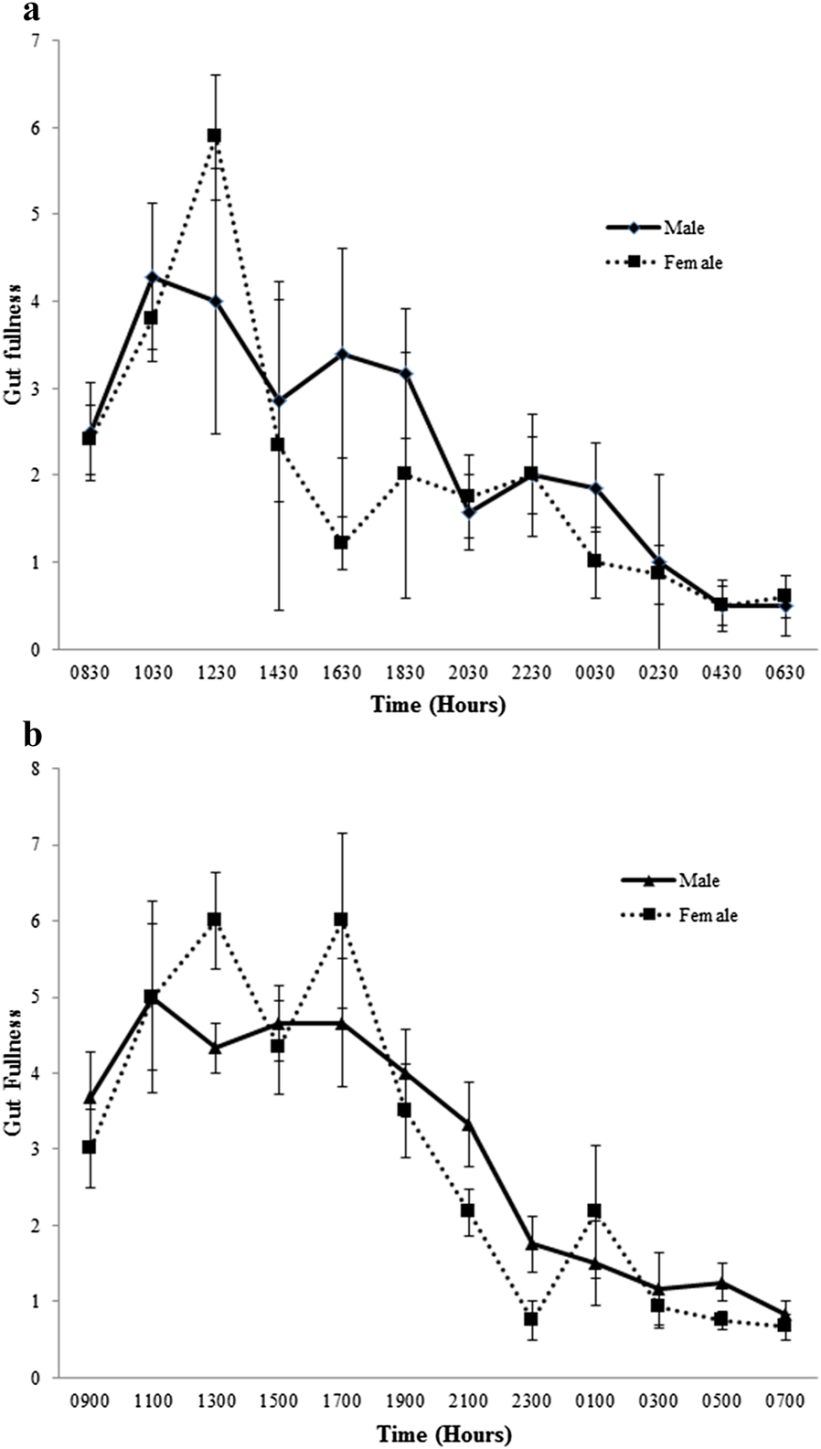
Fluctuations in gut fill (± SE) of *A. parvus* with time for females (depicted with square and hatched lines) and males (depicted with triangle and solid line) in **a** clay quarry pit and **b** reservoir

Gut fill results indicated that *A. parvus* is a diurnal feeder, as gut fill values were higher in daytime, but there was no difference in gut fill between the sexes and the diurnal patterns of feeding for both sexes in the two habitats were similar (Fig. 3).

**Fig. 4:**
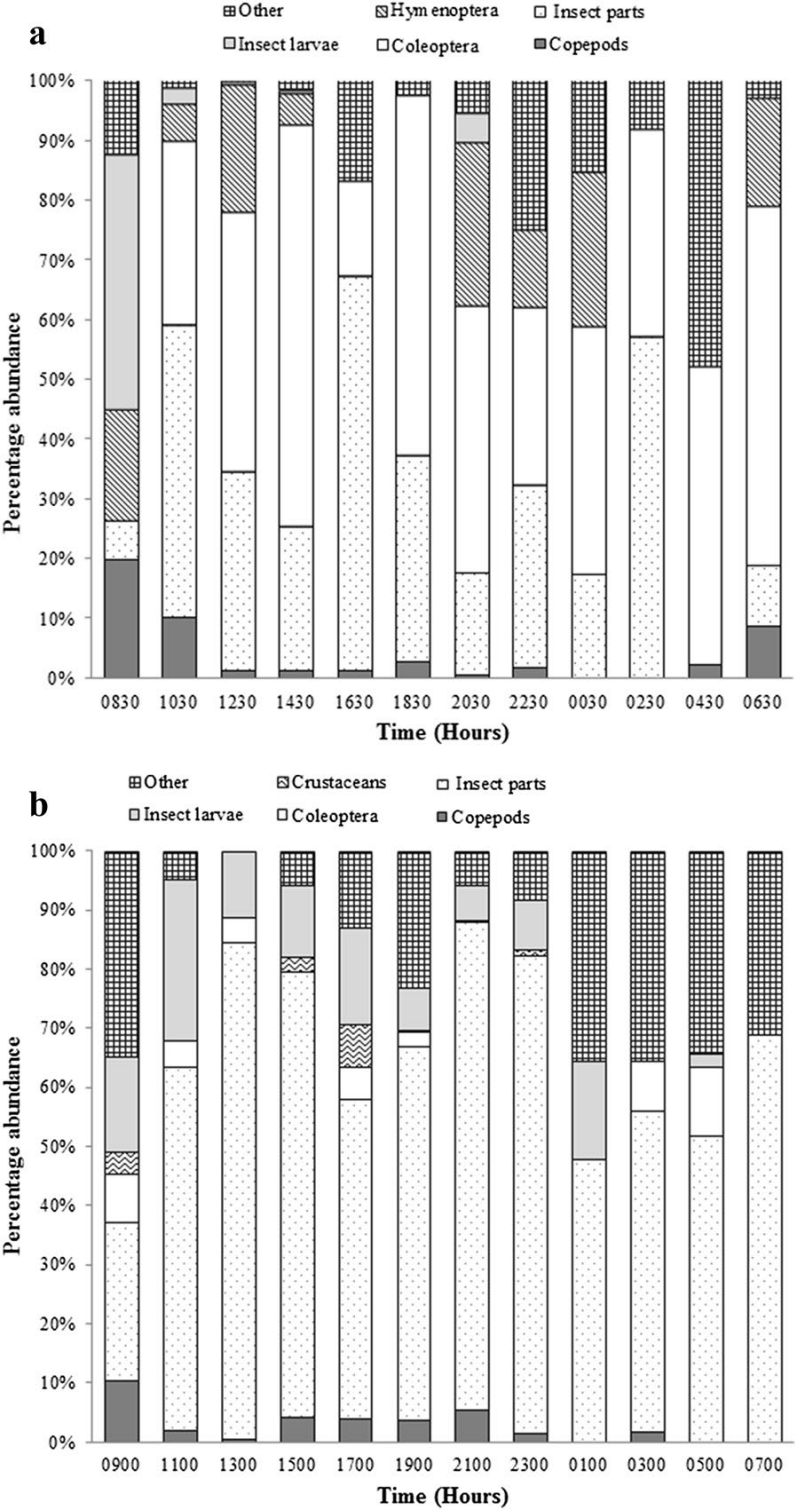
Diurnal variation of the percentage abundance of different food items in the gut contents of *A. parvus* sampled from the **a** clay quarry pit and **b** reservoir

For *A. parvus* in the clay quarry pit, total insect parts (unidentified insect parts, insect larvae, Hymenopterans and Coleopterans) ranged from 67.77% at 0830 h to 98.53% at 1230 h (Fig. 4a). In the reservoir, total insect parts (unidentified insect parts, insect larvae and Coleopterans) ranged from 50.94% at 0900 h to 99.53% at 1300 h (Fig. 4b).

### Diet of *P. reticulata*

Food items detected in the man-made canal and natural stream environments consisted of filamentous algae, detritus, copepods, insect parts and other items, which included plant parts, eggs and other unidentified materials. While copepods and filamentous algae were the main food items in the man-made canal, detritus represented the highest percentage of food items in the natural stream.

**Fig. 5:**
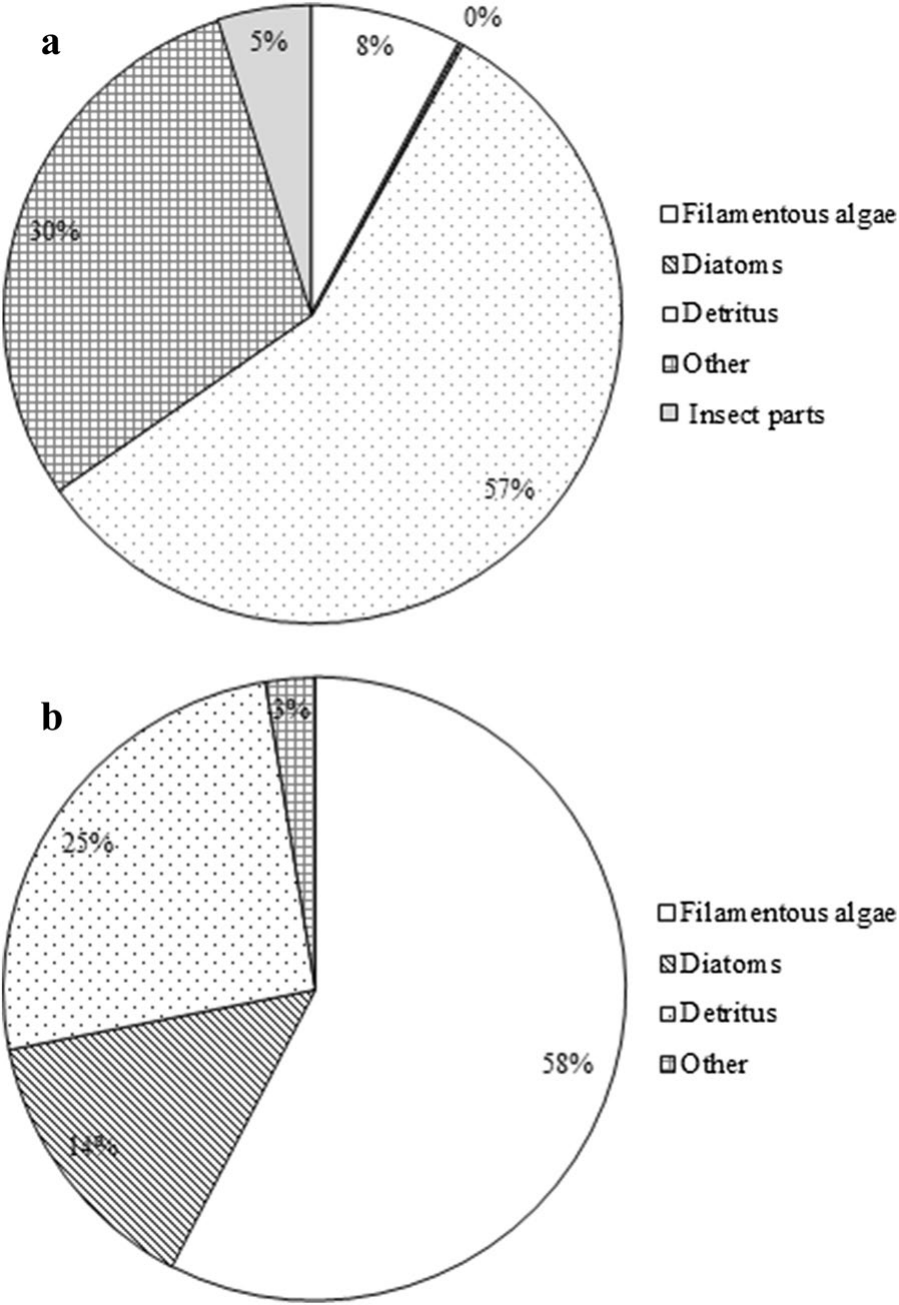
Percentage abundance of food items in the gut contents of *P. reticulata* collected from **a** natural stream and **b** man-made canal

The diet of *P. reticulata* in the man-made canal and natural stream environments consisted of filamentous algae, diatoms, detritus and other food items, which included plant parts, eggs and unidentified parts. In addition to those food items, approximately 5% of insect parts were also detected in fish collected from the natural stream (Fig. 5a). Whilst filamentous algae and detritus were the main food items in the gut in the man-made canal, detritus and other types of food items were the main items consumed in the natural stream (Fig. 5b).

**Fig. 6:**
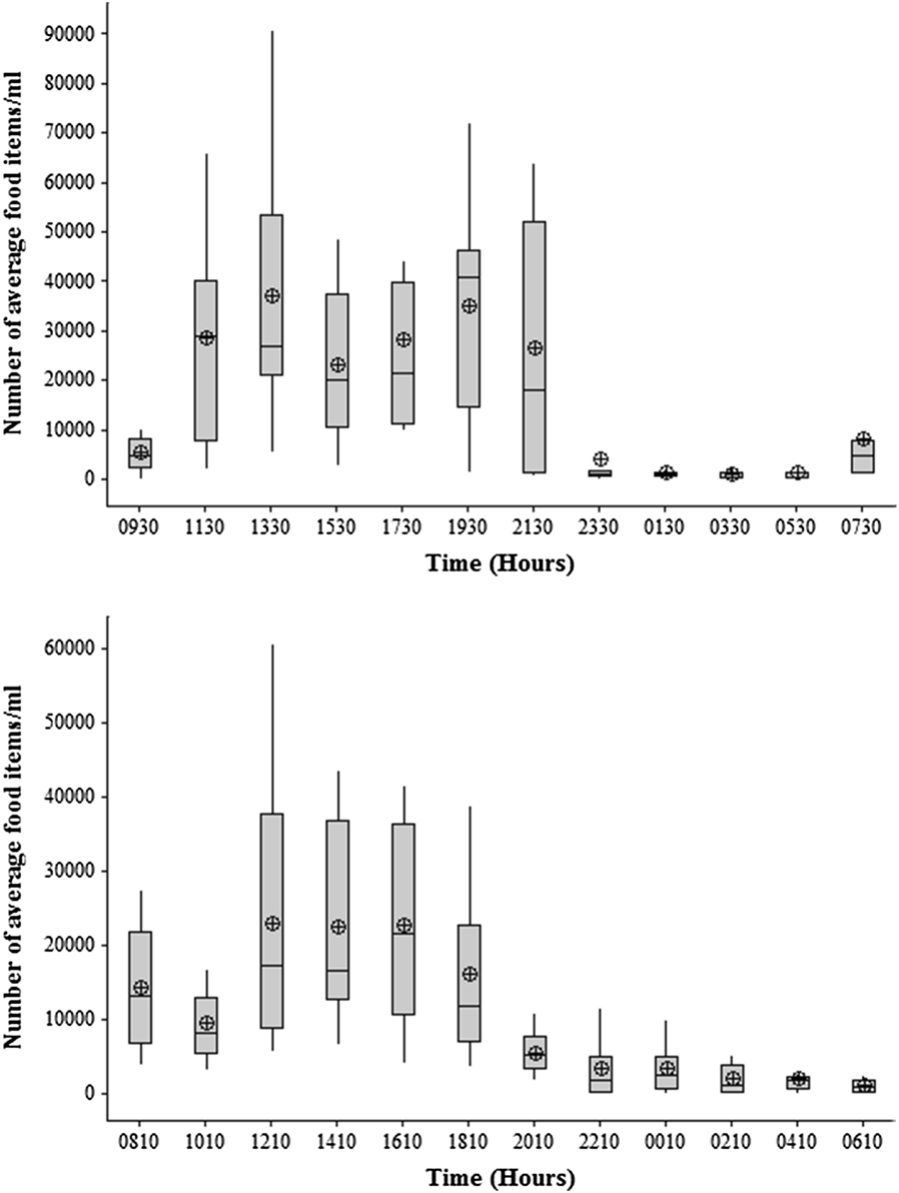
Fluctuations in number of mean food items with time in the gut contents of *P. reticulata* in **a** man-made canal and **b** stream. The box plot whiskers depict the range and circled crosses depict the mean number of food items

**Fig. 7:**
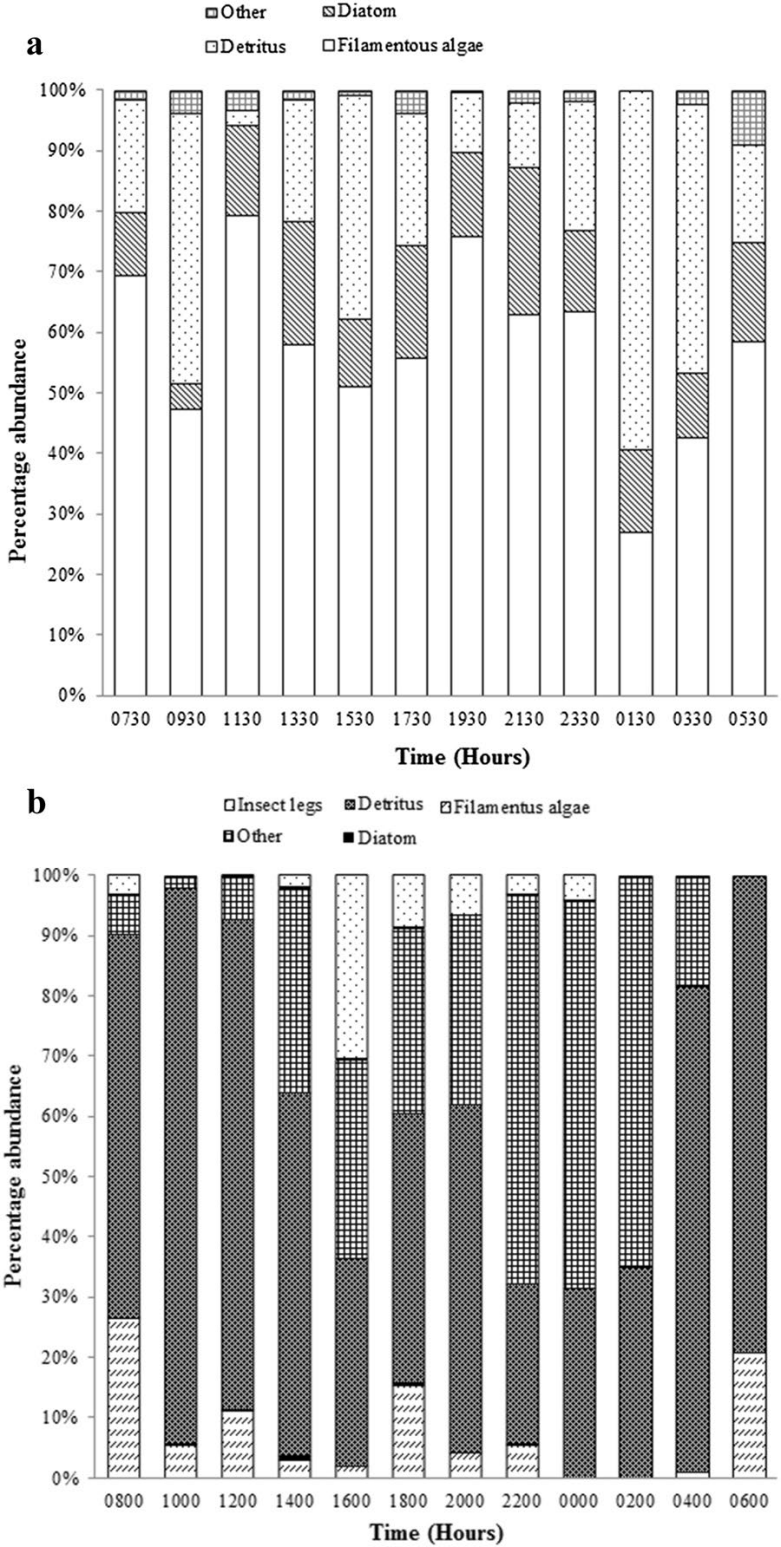
Diurnal variation of the abundance of different food items in the gut contents of *P. reticulata* sampled from **a** man-made canal and **b** natural stream

*Poecilia reticulata* had a higher mean number of food items in the gut in daytime compared to during the night and at dawn, in the man-made canal (ANOVA, *df*_11,128_, P < 0.005) (Fig. 6a). An almost identical pattern was seen in the natural stream (ANOVA, *df*_11,131_, P < 0.005) (Fig. 6b). Diatoms, filamentous algae and detritus accounted for over 90% of the diet (Fig. 7a).

In the natural stream, detritus was detected in the gut throughout the sampling period and ranged from 31% of gut fill at midnight to 92.29% at 1000 h. Detritus ranged between 22.65% of gut fill at 2200 h to 92.29% at 1000 h. Insect parts were present only in *P. reticulata* in the natural stream (Fig. 7b).

### Comparison between availability of food items and diet composition

**Fig. 8:**
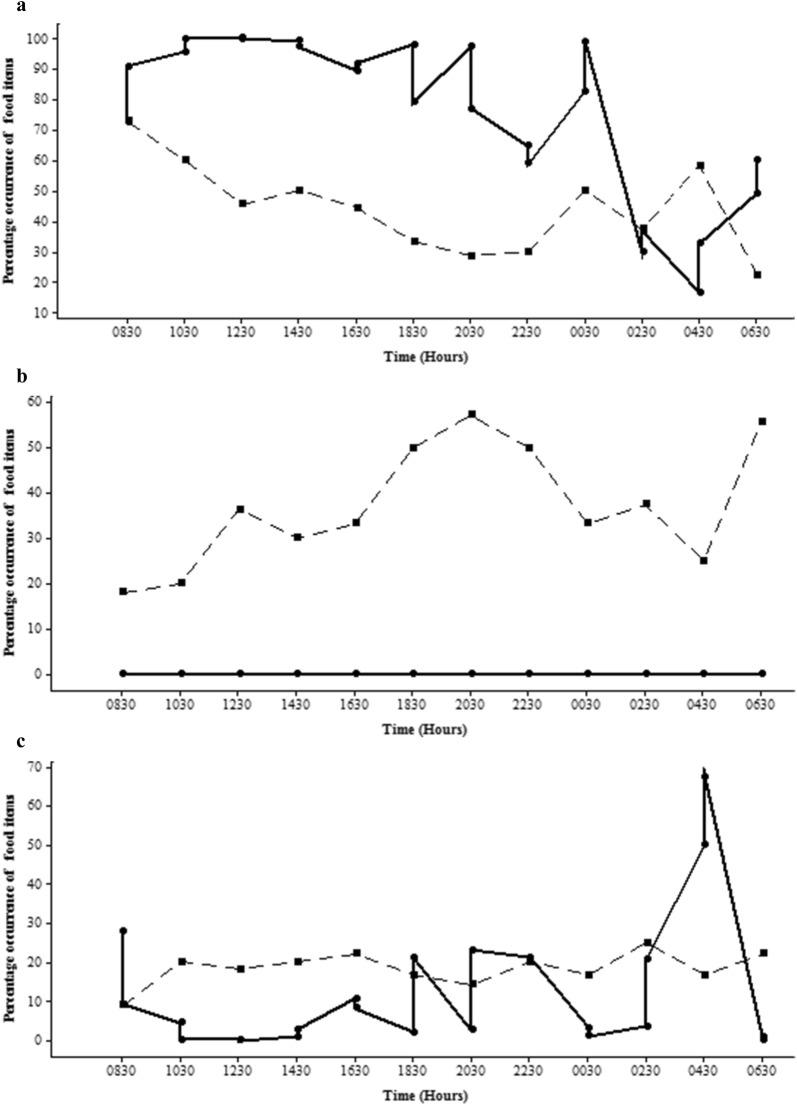
Diurnal variation of the percentage occurrence of **a** zooplankton and insect parts; **b** phytoplankton; **c** other types of food items in gut contents of *A. parvus* in the clay quarry pit at different time intervals (Gut-dipicted with circle and solid line; Environment-dipicted with square and hatched line)

**Fig. 9:**
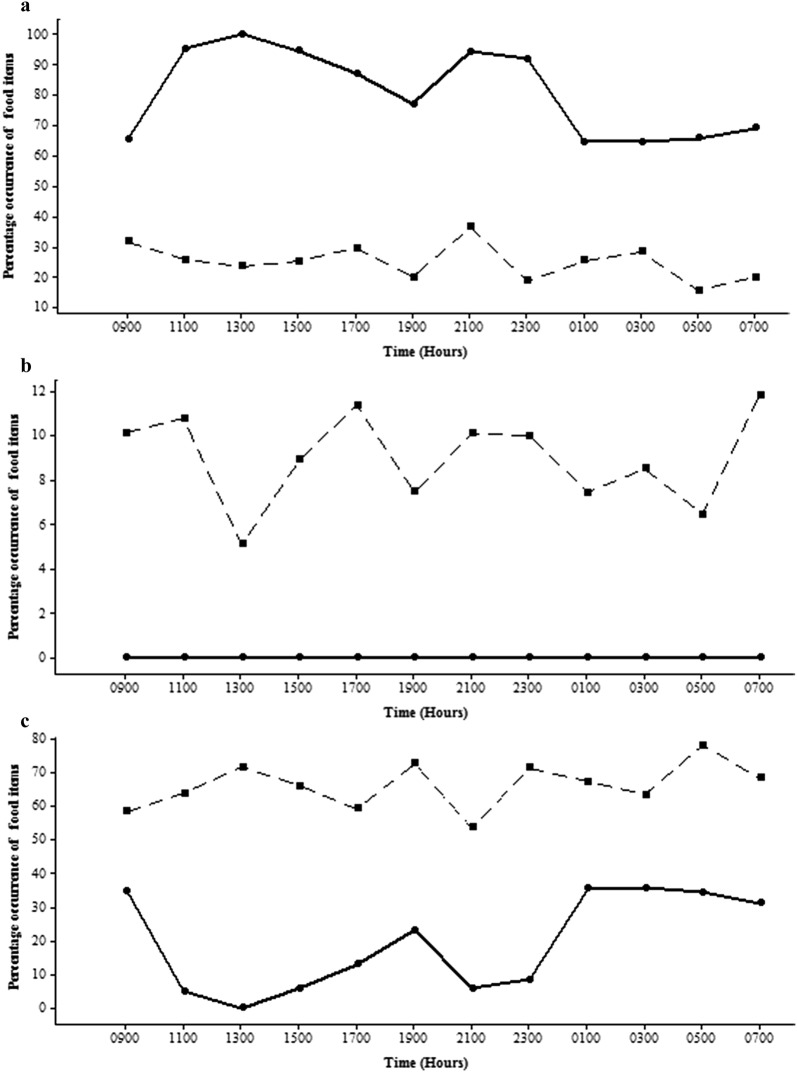
Diurnal variation of the percentage occurrence of **a** zooplankton and insect parts; **b** phytoplankton; **c** other types of food items in gut contents of *A. parvus* in the reservoir at different time intervals (Gut-dipicted with circle and solid line; Environment-dipicted with square and hatched line)

In the clay quarry pit *A. parvus* fed selectively on zooplankton and insect parts, with greater amounts consumed than were available in the environment (Fig. 8a) and avoided phytoplankton despite their greater availability (Fig. 8b). The same phenomenon was seen in the reservoir (Fig. 9a, b).

**Fig. 10:**
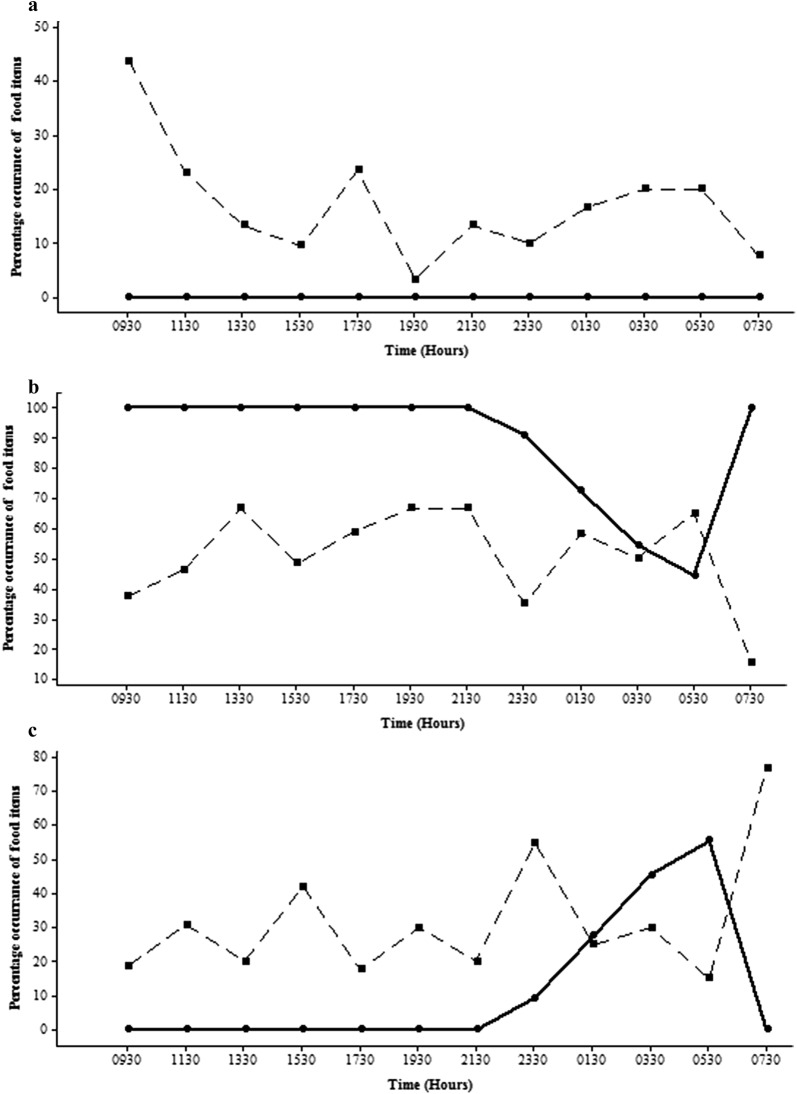
Diurnal variation of the percentage occurrence of **a** zooplankton and insect parts; **b** phytoplankton; **c** other types of food items in gut contents of *P .reticulata* in the man - made canal at different time intervals (Gut-dipicted with circle and solid line; Environment-dipicted with square and hatched line)

**Fig. 11:**
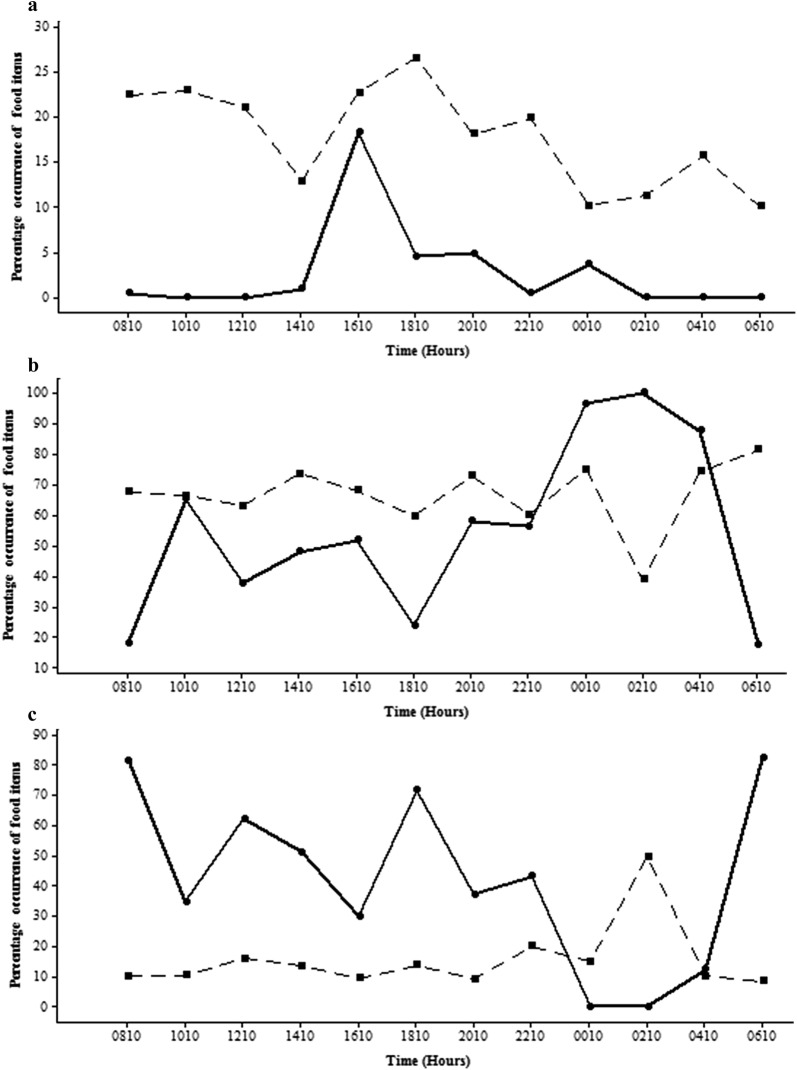
Diurnal variation of the percentage occurrence of **a** zooplankton and insect parts; **b** phytoplankton; **c** other types of food items in gut contents of *P. reticulata* in the natural stream at different time intervals (Gut-dipicted with circle and solid line; Environment-dipicted with square and hatched line)

The gut contents of *P. reticulata* comprised a significantly higher percentage of phytoplankton in both habitats (Figs. 10b and 11b). While the percentage of zooplankton and insect parts in the gut was almost zero, the percentage in the environment was comparatively higher (Figs. 10a and 11a) in the man-made canal (17.03% ± 10.51) and in the natural stream (2.7% ± 5.24).

Results of Electivity Index of Ivlev (1961) (Table 1) further confirmed that *A. parvus* had a higher selection over zooplanktons and insect parts in both the sites, while *P. reticulata* elected for phytoplankton.

**Table 1:** Results of the Electivity Index of *A. parvus* inhabiting reservoir and clay quarry pit and *P. reticulata* in man-made canal and stream

Species	Habitat	Zooplankton and insect parts	Phytoplankton	Other
*A. parvus*	Clay quarry pit	0.238**	− 1.000	− 0.411
	Reservoir	0.524**	− 1.000	− 0.588
*P. reticulata*	Man-made canal	− 1.000	0.264**	− 0.654
	Natural stream	− 0.804	0.299**	− 0.147

** Indicates the selection of food item

## Discussion

Contrary to the popular belief that *P. reticulata* is a larvivorus fish [9, 23], our results revealed them to be either planktivores or detritivores in the two new environments to which they have been introduced. However, throughout this study and irrespective of the environment, *A. parvus* showed a marked preference for insects and insect larvae over other available food items. Similar observations have been reported by others [24–26], suggesting that the idea that *P. reticulata* is a larvivorous fish is now questionable.

The reasons for diet differences could lie in the relationships between life history and diet. Several authors have confirmed that there is a strong association between the life history and diet of *P. reticulata* [26–28]. Zandonà et al. [26] further revealed that *P. reticulata* found in habitats with high predatory pressure feed primarily on invertebrates whilst under low predatory pressure; they fed primarily on detritus and algae. Additionally, under high predatory pressure, invertebrates with higher nutritional value were selected and consumed, but no such selection was observed under low predatory pressure, where consumption was decided by availability.

Introducing alien fish species can have many negative impacts on the native species [29, 30]. For instance, *Litoria aurea* [31], *Litoria dentate* [32], *Megalagrion* spp [33], *Edelia vittata* [34], *Bostockia porosa* [34], *Galaxias occidentalis* [34], *Rana muscosa* [35], *Limnodynastes ornatus* [36] and *Linderiella occidentalis* [37] are some of the native aquatic species that have been affected by introductions of exotic fish species in different parts of the world.

The two species introduced for mosquito control in Sri Lanka, *Gambusia affinis*, in 1940s and *Poecilia reticulata* in 1930s from Central America [17] are known invasives [7, 8]. According to current literature, *G. affinis* has already spread up to Northwestern [38] and Western [39] parts of the country. *P. reticulata* is also recorded to have spread up to northwestern [38], western [39] and southern [40] provinces. For the current study, the species was collected from Salgala in Sabaragamuwa province, the foot hills of central mountain massif.

It is, therefore, suggested that future plans for any type of biological control using fish should give priority to native species where possible. Use of native fish species rather than exotics has been encouraged [41, 42] and a number of attempts have been made to use them [13, 14, 43, 44]. This strategy can be justified by the diet composition results for *P. reticulata* in the current study. Further, *P. reticulata* shown a detritivorous consumption in the wild in certain habitats. So, although experimentally this species is capable of consuming insect larvae [45], its use as a biological control can be more harmful than beneficial.

Knowledge of the predatory pressure of a given habitat can be beneficial when introducing *P. reticulata* as larvivorous fish in a particular body of water. By comparing the current findings with those of Zandonà et al. [26], it could be proposed that the natural stream had more predatory pressure than the man-made canal. The current study did not find any aquatic environment where both the species were present naturally, hence the outcomes from this study do not allow comparison of their diet choices when they are co-existing.

## Conclusion

The use of natives over exotics as biological control agents is gaining popularity and this is partly due to issues to do with the invasiveness of exotics. As *A. parvus* fed during the day as well as during the night, there could be more opportunity for consumption of insect larvae, as well as egg-laying adults, since they are surface feeders. As *P. reticulata* is already considered to be an invasive species, *A. parvus* should be studied further to assess its efficacy as a controlling agent for insects, especially mosquitoes. However translocation of natives such as A. *parvus* should be done with care as there are distinct species assemblages even between different water sheds of a given country. *A. parvus* is a selective feeder and, compared to *P. reticulata,* showed a preference for an insectivorous diet in the habitats studied.

## Additional file

**Additional file 1.** **Plate S1.** Food items detected in the sampled clay quarry pit and reservoir a: Filamentous algae (×10); b: Detritus (×4); c: Plant part (×4); d: Copepod (×4); e: Insect leg (×4)**. Plate S2.** Food items identified in the gut of *A. parvus* inhabiting the sampled clay quarry pit and reservoir**—**a: Coleopteran larvae (×4); b: Adult Coleopteran (×4); c: Part of a Coleopteran (×4); d:e:f: Hymenopterans (×4); g:h:Insect larval stages(x); i:Copepod. **Plate S3**. Food items detected in the sampled natural stream and man-made canal**—**a: Filamentous algae (×10); b: Detritus (×4); c: Eggs (×10). **Plate S4.** Food items identified in the gut of *P. reticulata* inhabiting the sampled man-made canal—Microscopic field consisted of filamentous algae and diatoms(×4).
