# Supplementary material for: A comparison of the larvivorous habits of exotic Poecilia reticulata and native Aplocheilus parvus
Source: BMC Ecol. 2018 Aug 14;18:25. doi: 10.1186/s12898-018-0180-1 (PMC6092854; doi:10.1186/s12898-018-0180-1)
Supplement: Supplementary file 1 — Additional file 1. Plate S1. Food items detected in the sampled clay quarry pit and reservoir a: Filamentous algae (×10); b: Detritus (×4); c: Plant part (×4); d: Copepod (×4); e: Insect leg (×4). Plate S2. Food items identified in the gut of A. parvus inhabiting the sampled clay quarry pit and reservoir—a: Coleopteran larvae (×4); b: Adult Coleopteran (×4); c: Part of a Coleopteran (×4); d:e:f: Hymenopterans (×4); g:h:Insect larval stages(x); i:Copepod. Plate S3. Food items detected in the sampled natural stream and man-made canal—a: Filamentous algae (×10); b: Detritus (×4); c: Eggs (×10). Plate S4. Food items identified in the gut of P. reticulata inhabiting the sampled man-made canal—Microscopic field consisted of filamentous algae and diatoms(×4). [file 12898_2018_180_MOESM1_ESM.docx]

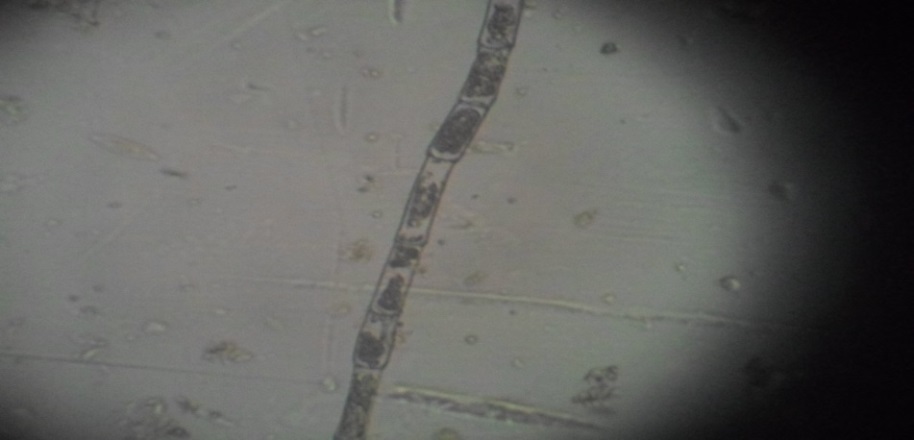

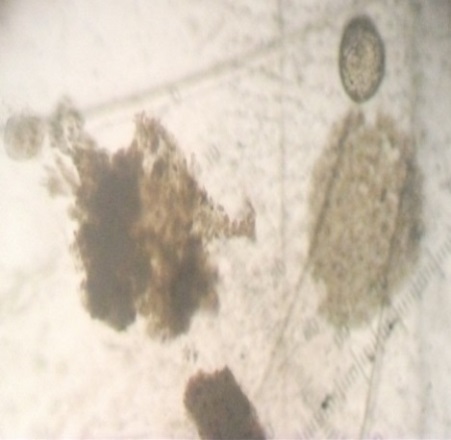

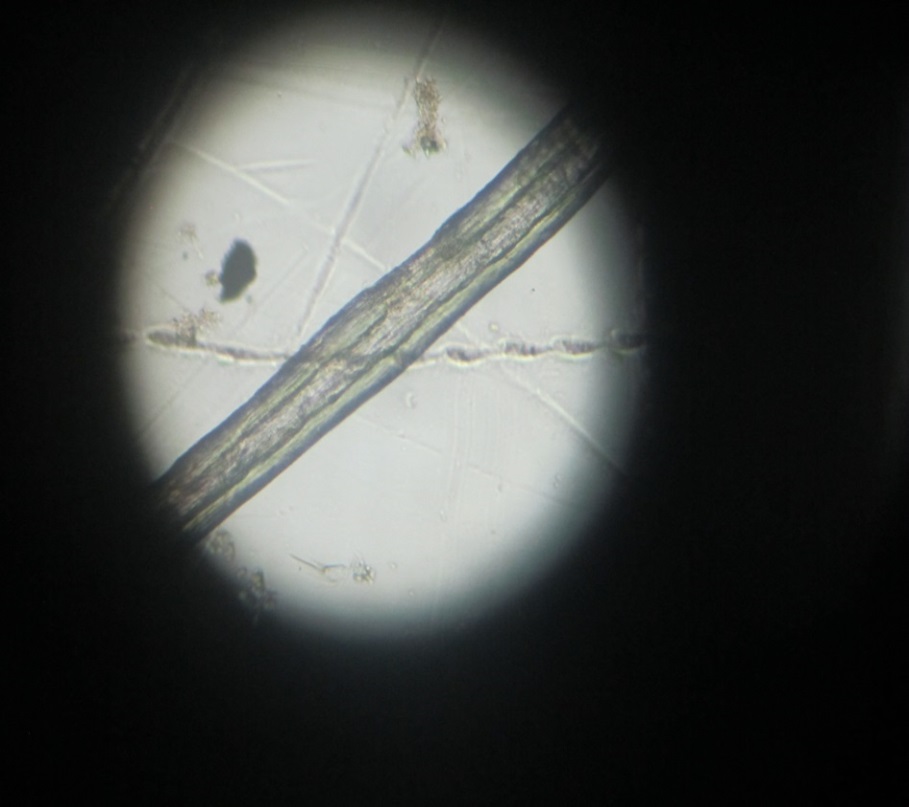


a b c


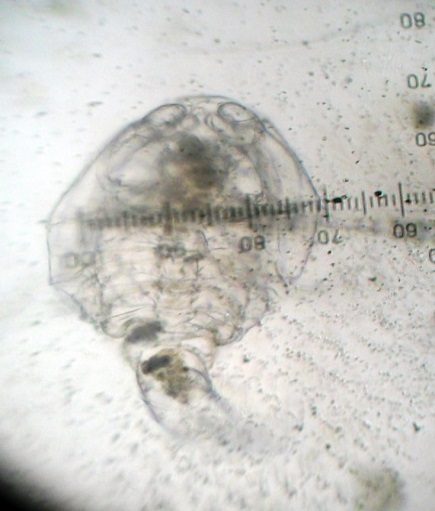

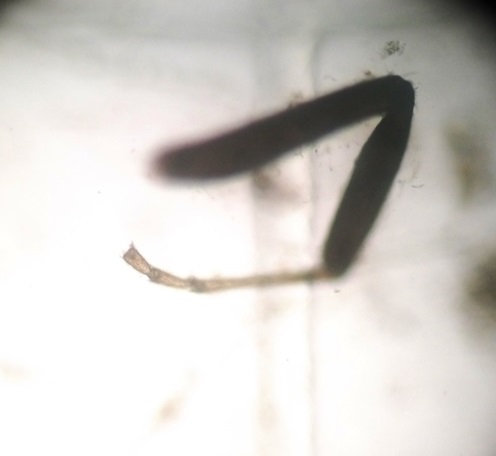


d e

Plate S1. Food items detected in the sampled clay quarry pit and reservoir a: Filamentous algae (x10); b: Detritus (x4); c: Plant part (x4); d: Copepod (x4); e: Insect leg (x4)


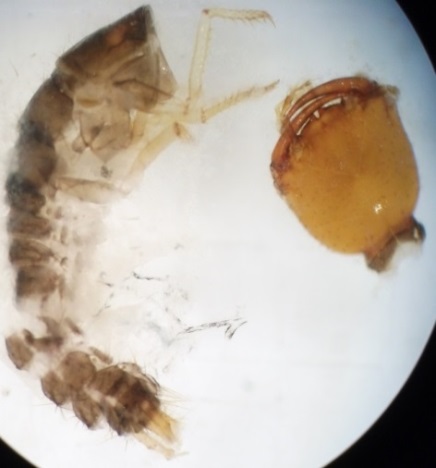

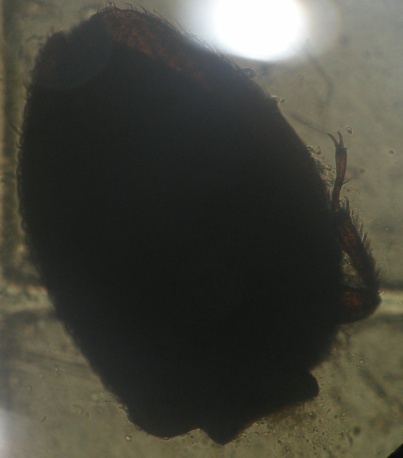

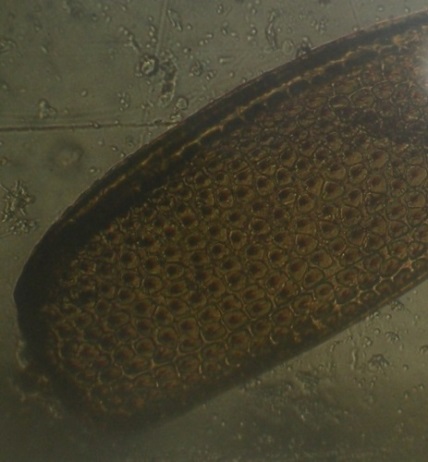


a b c


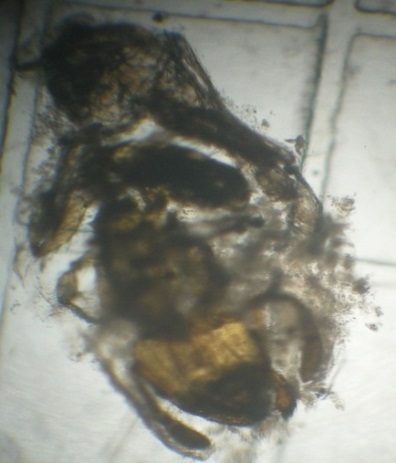

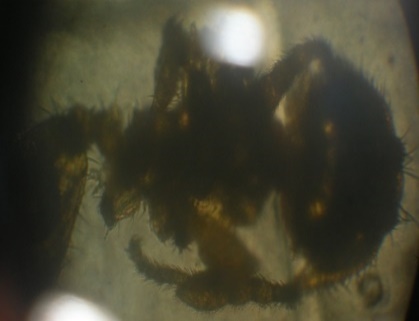

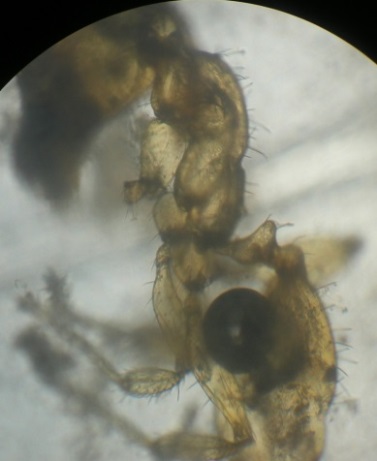


d e f


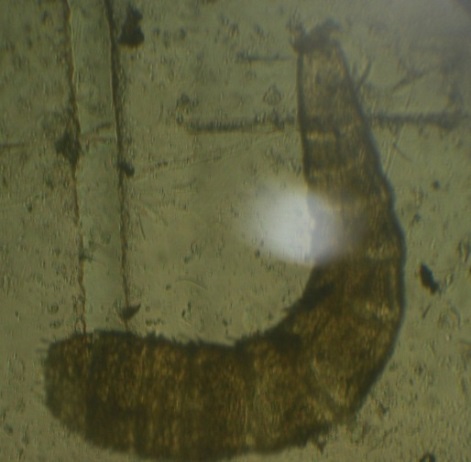

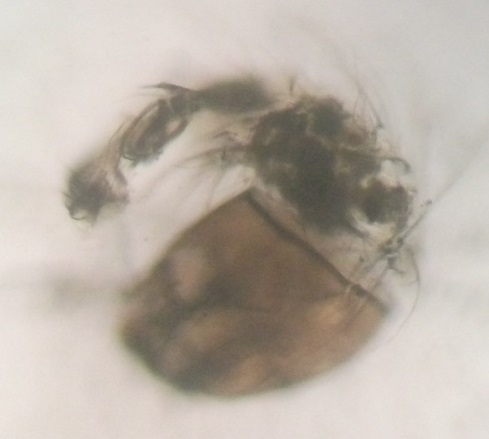

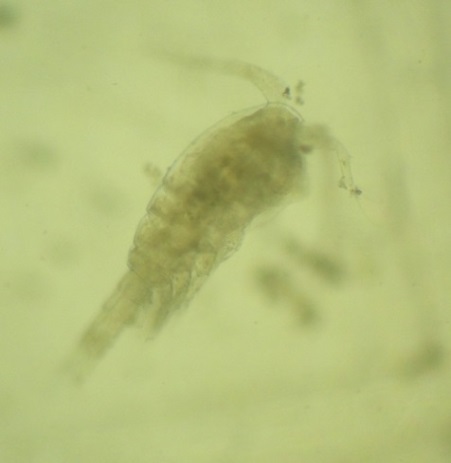


g h i

Plate S2. Food items identified in the gut of *A. parvus*  inhabiting the sampled clay quarry pit and reservoir – a: Coleopteran larvae (x4); b: Adult Coleopteran (x4); c: Part of a Coleopteran (x4); d:e:f: Hymenopterans (x4); g:h:Insect larval stages(x); i:Copepod


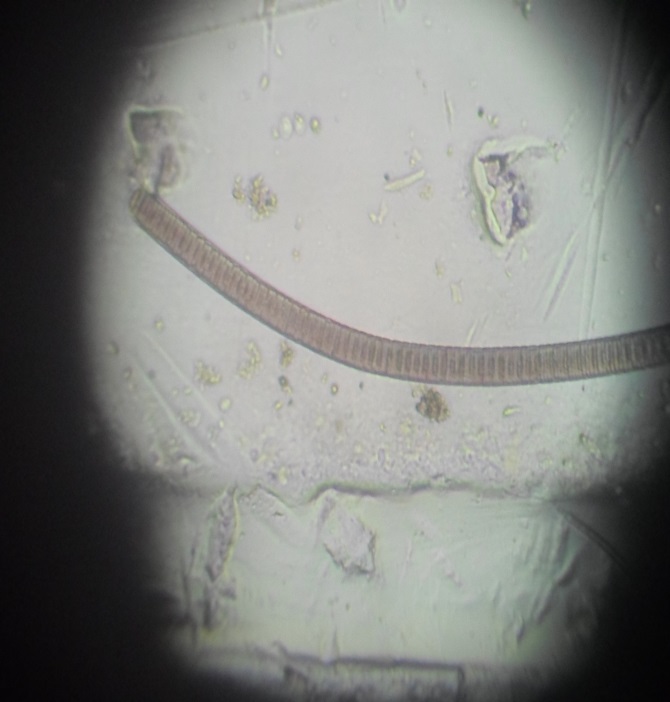

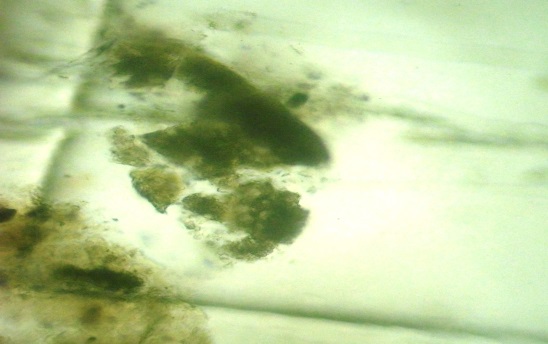

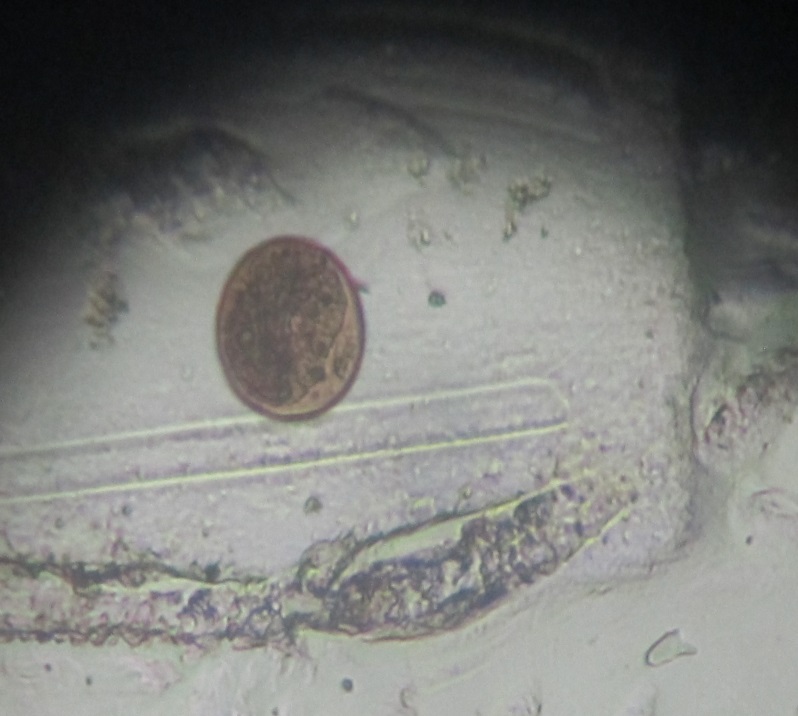


a b c

Plate S3. Food items detected in the sampled natural stream and man-made canal - a: Filamentous algae (x10); b: Detritus (x4); c: Eggs (x10)


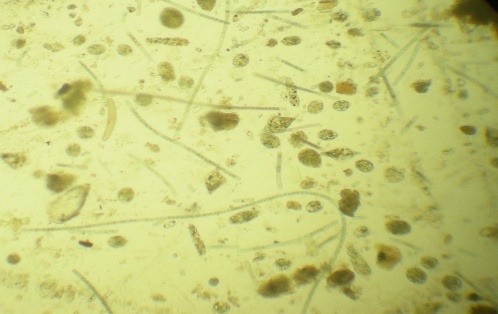


Plate S4. Food items identified in the gut of *P. reticulata* inhabiting the sampled man- made canal - Microscopic field consisted of filamentous algae and diatoms(x4)
